# A Rare Case of Non-immune Hemolytic Anemia in a Stage IV Breast Cancer Patient Treated With Capecitabine

**DOI:** 10.7759/cureus.24921

**Published:** 2022-05-11

**Authors:** Hussain Hussain, Aya Fadel, Victor Guardiola, Sergio Rodriguez

**Affiliations:** 1 Internal Medicine, Universal Health Clinic, Miami, USA; 2 Internal Medicine, Hackensack Meridian Ocean University Medical Center, Brick Township, USA; 3 Oncology, Baptist Health South Florida, Miami, USA; 4 Internal Medicine, Nova Southeastern University Dr. Kiran C. Patel College of Osteopathic Medicine, Fort Lauderdale, USA

**Keywords:** capecitabine side effects, her 2 negative, er-pr positive, adjuvant tamoxifen, docetaxel, anemia and hyperbilirubinemia, metasatic metaplastic breast cancer, capecitabine, non-immune hemolytic anemia

## Abstract

Incremental changes in the diagnosis of breast cancer leave drastic impacts on patients. There are detrimental shifts in cost, psychological disorders in terms of depression, and morbidities. Stage IV breast cancer has a high mortality rate and was afflicting our patient who was diagnosed with metastatic breast cancer estrogen receptor/progesterone receptor (ER/PR) positive, human epidermal growth factor receptor 2 (HER-2) neo negative, and low Ki-67. Among the various management modalities and effective treatments, capecitabine was selected because of its benefits; however, there are several commonly known adverse effects when using capecitabine including non-immune hemolytic anemia, a very rare and unexpected side effect despite the many research and clinical trials performed. Immune mediate or Coombs positive hemolytic anemia was reported with the usage of capecitabine, but this patient developed Coombs negative or non-immune hemolytic anemia. Capecitabine, a form of fluoropyrimidine, hypothetically affects the erythrocyte membrane structure resulting in the destruction of these cells. Additionally, discontinuation of capecitabine in the patient led to the resolution of the condition; this made us more aware of the precise diagnosis, also considering that the bone marrow biopsy came back negative.

## Introduction

Breast cancer is very common with approximately one in every 10 females developing breast cancer during their lifetime [1]. Fortunately, death rates due to breast cancer have been decreasing over the last few decades, in part due to earlier diagnosis via diagnostic imaging modalities such as mammograms and ultrasounds and other improvements in effective treatments [1]. However, breast cancer is still the second-most common cancer affecting women aside from skin cancer [1,2]. Moreover, breast cancer can metastasize to various organs including liver, lung, and bone [2]. Bone marrow infiltration by malignant cells can lead to devastating consequences including severe pancytopenia [2].

Hemolytic anemia is the destruction of red blood cells via various mechanisms [2]. Capecitabine, which is used for breast cancer treatment among other cancers can lead to hemolytic versus non-hemolytic anemia as a rare side effect [2-4]. Docetaxel is commonly used in breast cancer for both prevention and treatment and can induce myelodysplasia or secondary malignancies such as leukemia or lymphoma as a form of a rare side effect after months to years from exposure [2-4].

## Case presentation

A 37-year-old female (G3P2 A1) presented to the oncology clinic after being diagnosed with locally advanced right breast cancer (clinical T3N1), estrogen receptor/progesterone receptor (ER/PR) positive, human epidermal growth factor receptor 2 (HER-2) neo negative, low Ki-67. Genetic tests were conducted but resulted in negative results for the *BRCA1* and *BRCA2* genes. Given her large tumor size and lymph node positivity, the decision was made to proceed with neoadjuvant chemotherapy in the form of docetaxel and cyclophosphamide every three weeks for six rounds. After a partial response to treatment, it was decided to proceed with a bilateral nipple-sparing mastectomy where a final pathology report revealed a residual 1.4 cm invasive ductal carcinoma on the right breast, moderately differentiated and margin free. A total of 14 lymph nodes were removed, six of them being sentinel in which one lymph node had macrometastases, and four lymph nodes had micrometastases. Given the above, she received adjuvant radiation therapy and anti-hormonal therapy, consisting initially of goserelin and tamoxifen. Once serological proof of menopause was confirmed, the patient was swapped to anastrozole therapy.

Despite the patient being responsive to adjuvant endocrine therapy, three years later, in April of 2019, the patient was found to have relapsed. This was confirmed after abdominal discomfort was reported and a liver function test was performed where abnormalities were noted. A biopsy provided proof of liver metastasis disease with ER/PR positive and HER-2 neo negative. At the same time, the disease was found in the bone, liver, and lymph nodes as well as peritoneal carcinomatosis. The patient was treated with first-line single-agent paclitaxel, achieving a partial response. After three months, a decision was made to change to second-line palbociclib and fulvestrant with continued response. By the end of 2019, she underwent a bilateral oophorectomy; unfortunately, soon after, the patient had a progression of the disease, leading to the discontinuation of palbociclib and fulvestrant and commencement of single-agent paclitaxel.

**Figure 1:**
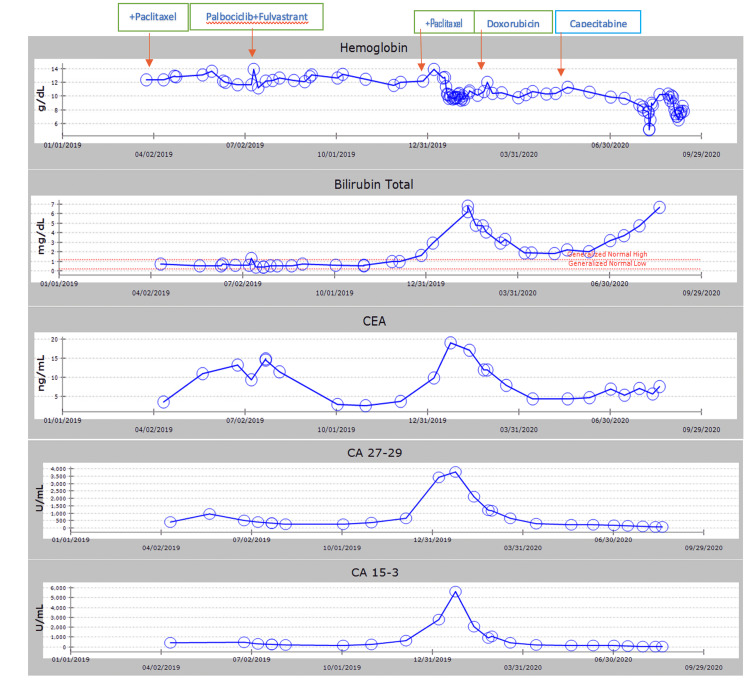
Targeted treatments initiated and laboratory values CEA: carcinoembryonic antigen; Ca 27-29: cancer antigen 27-29; Ca 15-3: cancer antigen 15-3

In January 2020, soon after starting paclitaxel, the patient required hospitalization due to a worsening liver function test and worsening tumor burden. The patient was no longer a candidate for paclitaxel therapy as evidenced by the worsening bilirubin and liver function tests. However, the decision was made to start liposomal doxorubicin in which a partial response was achieved with the normalization of bilirubin as LFTs were trending down and tumor markers were improving. The timeline and summary of laboratory and imaging reports are given in Table 1. Figure 1 shows the relation between targeted treatments and laboratory results.

**Table 1:** Timline and summary of laboratory tests and imaging performed FDG: fluorodeoxyglucose; PET: positron emission tomography; LFT: liver function test

Imaging		LFT	Hemoglobin	Tumor markers
April 2016 to January 2019: no evidence of metatstatsis		Normal	Normal	Tumor markers were high.
April 2019 to June 2019: metastatic disease involving multiple organ		High	Normal	Tumor markers were high
July 2019 to September 2020: findings consistent with posttreatment changes in the liver with no FDG uptake		Slightly up	Normal	Tumor markers started to trend down
October to November, 2019: new FDG avid metastatic L5 vertebral body. Findings consistent with posttreatment changes in the liver with no FDG uptake		Slightly up	Normal	Tumor markers continue to trend up again
December 2019: Abdominal MRI revealed a pseudocirrhotic appearance		Very high to trending down later to reach the upper normal to slightly high by March 2020	Normal	Tumor markers were very high
January to April, 2020: CT scan showed a new left femoral mass		Normal to slightly high	Low	Tumor markers were very high and start to trend down by February 2020
May 2020 to June 2020: PET scan revealed a progression of bone metastasis, severe liver cirrhosis, and liver parenchyma, which may be related to pseudocirrhosis from underlying malignant infiltration or posttreatment changes. Large bilateral pleural effusions and moderate to large volume ascites likely related to portal hypertension and fluid overload.		Slightly high to normal	Low	Tumor markers were high and started to trend down by June 2020
July 2020 to August 2020: Abdominal MRI revealed advanced liver cirrhosis, pseudocirrotic, right lobe metastasis and peritoneal carcinomatosis.	Slightly high to normal		Low	Tumor markers were slightly up to normal

In April 2020, after three months of treatment with liposomal doxorubicin, the decision was made to change to single-agent capecitabine. The patient took a vacation for three months but, unfortunately in August 2020, she was admitted to the hospital for cellulitis over the left thigh and was found to have profound anemia, thrombocytopenia, and elevation of bilirubin. At that point in time, the suspicion of hemolysis due to treatment with capecitabine was entertained. The patient had elevated levels of reticulocyte and lactate dehydrogenase (LDH) with low haptoglobin levels. A Coombs test was performed but came back negative; given the fact that it was not proven to be an autoimmune-mediated hemolytic process, the decision was to rule out potential bone marrow infiltration. A bone marrow aspiration/biopsy was obtained while capecitabine was on hold, and the results led to suspected early myelodysplasia with no evidence of chromosomal abnormalities and normal cytogenetics. The patient received supportive transfusions while no further treatment of the patient’s breast cancer was pursued as the disease was presumed to be somewhat under control and focused on supportive care regarding pseudocirrhosis.

## Discussion

Capecitabine is an oral form of fluoropyrimidine prodrug that is metabolized to 5-fluorouracil (5FU) in three steps [3,4]; initially, capecitabine is hydrolyzed by carboxylesterase to form 5 deoxyfluorocytidine, which then undergoes a second metabolism by cytidine deaminase to produce 5 deoxy-5 fluorouridine, which is considered to be active in cancer cells as well as liver cells [3,4]. Lastly, the 5-deoxy-5-fluorouridine is converted within the tumor cells into 5FU via thymidine phosphorylase [3,4]. Eventually, DNA synthesis would be inhibited [3,4].

We present a very complex and challenging clinical case of a young female with metastatic breast cancer receiving palliative treatment. While the patient’s disease was controlled with the use of capecitabine, there was a development of cytopenia with suspicion of hemolysis leading to further workups. This showcases the usual complexity in treating patients with medications that have both acute and chronic side effects. This particular case was rewarding, partly because the bone marrow biopsy excluded the possibility of bone marrow infiltration as a potential etiology behind the development of cytopenia. However, the evidence of myelodysplasia morphologically made us wonder whether the chemotherapy in the form of docetaxel [4], given three years prior for prevention, could have led to implications at the time she received capecitabine despite no evidence of cytogenetic abnormalities with particular attention to chromosomal alteration in chromosomes five and seven. We concluded that the likely and probable combination of underlying myelodysplasia, not seen while on liposomal doxorubicin, complicated by the use of capecitabine in a patient with chronic liver disease from underlying liver metastatic disease could be the explanation for her clinical scenario.

Despite the reputation capecitabine has of potentially resulting in a Coombs positive autoimmune hemolytic anemia, we could not prove there was a correlation since her Coombs test came back negative under a hemolytic process, where some were cofounded by the profound liver dysfunction that the patient developed. The patient discontinued capecitabine and her condition improved after eight weeks along with trending down of the bilirubin level, which provided a clear explanation of her condition. Additionally, the early myelodysplastic changes return to normal after approximately three months, and the liver function test did not improve due to secondary liver lesions from metastatic breast cancer. Vitamin levels were within the normal range. Given that, we hypothesized that the destruction of erythrocytes occurred due to the effect of fluoropyrimidine on the erythrocyte cell membrane [5].

## Conclusions

Chemotherapy is widely used in the treatment of breast cancer, despite known side effects, because of its efficacy. The use of doxorubicin often leads to myelodysplasia, a potentially devastating phenomenon. This often leads the patient to continue to need further treatment to control the underlying metastatic breast cancer as it imposes challenges for further therapeutic intervention. Additionally, although capecitabine is known to rarely cause hemolysis, one should rule out the possibility of bone marrow infiltration or additional etiologies accounting for acute anemia in the context of confounding analysis in a patient with a high burden of the disease. For this, typical laboratory analyses including LDH, haptoglobin, and reticulocytes need to be interpreted.
